# Cohort Profile: TRacing Etiology of Non-communicable Diseases (TREND): Rationale, Progress and Perspective

**DOI:** 10.1007/s43657-024-00196-4

**Published:** 2024-10-16

**Authors:** Hui-Ying Ren, Ying Lv, Bei-Ning Ma, Chang Gao, Hong-Mei Yuan, Hai-Hong Meng, Zheng-Qian Cao, Ya-Ting Chen, Yan-Xi Zhang, Yu-Ting Zhang, Wei Liu, Yu-Ping Fan, Meng-Han Li, Yu-Xuan Wu, Zhuo-Yue Feng, Xin-Xin Zhang, Zhen-Jian Luo, Qiu-Yi Tang, Anke Wesselius, Jian Chen, Hong-Xing Luo, TREND Cohort Study, Qi-Rong Qin, Lianmin Chen, Evan Yi-Wen Yu

**Affiliations:** 1 Department of Epidemiology & Biostatistics, School of Public Health, Southeast University, Nanjing, 210009 China; 2 Key Laboratory of Environmental Medicine and Engineering of Ministry of Education, School of Public Health, Southeast University, Nanjing, 210009 China; 3 School of Public Health, Wannan Medical College, WuHu, 241002 China; 4 Department of Cardiology, The First Affiliated Hospital of Nanjing Medical University, Nanjing Medical University, Nanjing, 210029 China; 5 Department of Gastroenterology, Changzhou Medical Center, The Third Affiliated Hospital of Nanjing Medical University, Nanjing Medical University, Changzhou, 213164 China; 6 School of Life Sciences, Westlake University, Hangzhou, 310024 China; 7 Health Commission of YuShan District, Ma’anshan, 243000 China; 8 Department of General Surgery, Xinhua Hospital Affiliated to Shanghai Jiao Tong University School of Medicine, Shanghai, 200092 China; 9 Department of Epidemiology, CAPHRI Care and Public Health Research Institute, Maastricht University, Maastricht, 6229ER The Netherlands; 10 School of Nutrition and Translational Research in Metabolism, Maastricht University, Maastricht, 6229ER The Netherlands; 11 Ma’anshan Center for Disease Control and Prevention, Ma’anshan, 243011 China; 12 Institute for Surgical Research, Oslo University Hospital, 0424 Oslo, Norway; 13 Department of Physiology, Maastricht University, Maastricht, 6211LK The Netherlands

**Keywords:** Non-communicable diseases, Multi-omics, Deep phenotyping, State-of-art techniques, Prospective design

## Abstract

The TRacing Etiology of Non-communicable Diseases (TREND) cohort is a prospective longitudinal cohort and biobank that is mainly based in Ma’anshan, Anhui Province, China. The primary aim of the study is to decipher comprehensive molecular characterization and deep phenotyping for a broad spectrum of chronic non-communicable diseases (NCDs), which focuses on providing mechanistic insights with diagnostic, prognostic and therapeutic implications. The recruitment was initiated in 2023 and is expected to complete in 2025 with 20,000 participants originated from urban and rural area. In the first phase, 3360 participants were recruited. Follow-up visits are scheduled annually and intervally for a total of 30 years. The cohort includes individuals aged over 18 years. Two participants with first-degree linkage were recruited from a household. The age distribution of recruited participants was stratified into four categories: 18–45, 45–55, 55–65, and ≥65 years, aligning with the population proportions of Ma’anshan. Meanwhile, the gender distribution was controlled by pairing men and women from the same household. Data collected at baseline includes socio-economic information, medical history, lifestyle and nutritional habits, anthropometrics, blood oxygen, electrocardiogram (ECG), heart sound, as well as blood, urine and feces tests results. Molecular profiling includes genome, proteome, metabolome, microbiome and extracellular vesicles -omics. Blood, urine and fecal samples are collected and stored at −80 °C in a storage facility for future research.

**Supplementary Information:**

The online version contains supplementary material available at 10.1007/s43657-024-00196-4.

## Introduction

Chronic non-communicable diseases (NCDs) such as cardiovascular diseases, metabolic dysfunction, cancer, respiratory diseases, degenerative diseases, and psychiatric diseases have emerged as predominant contributors to life-threatening conditions and global mortality (Collaborators and Ärnlöv 2020; WHO 2023). These impairments are multi-factorial in origin, often involving complex mechanisms that pose challenges in terms of prevention, treatment and prognostic care. While large-scale observational prospective studies have been undertaken to unravel causality, various essential factors beyond those with mechanistic explanation, remain undetermined due to phenotypic variance and limitation of general observational data. Furthermore, with the rapid development of biotechnology and mobile health (mHealth) (Istepanian et al. 2007), there is a need for well-characterized study designs incorporating in-depth multi-layered data to bridge the gaps between observational findings and potential mechanistic insights concerning NCDs.

In the past years, a shift in the pattern of NCDs pattern has been observed, transitioning between urban and rural area in China (Peng et al. 2023). However, few studies focused on the impact of urbanization on NCDs. The city of Ma'anshan, situated in the lower reaches of the Yangtze River, emerges as an ideal location for assessing the effects of urbanization on NCDs. Its unique characteristic of having a resident population evenly distributed between urban and rural areas makes it particularly well-suited for studying the evolution of NCDs in the context of urbanization. Given that well-known iron and steel industries inner city, Ma'anshan has built a multi-located environmental surveillance system with daily monitoring on air, sewage, noise, light, and soil, offering valuable opportunities to investigate the health implications of environmental pollution.

A large body of evidence suggests a familial aggregation of many NCDs (Rasooly et al. 2019). However, there is notable heterogeneity in the risk profile of NCDs due to the interplay between environmental/lifestyle and genetic effects (Cooper 2003; Wang et al. 2023). Recognizing this complexity, the TRacing Etiology of Non-communicable Diseases (TREND) cohort, has adopted a unique approach by recruited participants as a pair (i.e., n = 2) from each household with first-degree linkage. This strategy is designed to enhance our understanding of gene-environment/lifestyle interactions in the development of NCDs. In summary, the TREND cohort aims to compile detailed epidemiological data by integrating extensive phenotyping, including a broad range of determinants (including extensive -omics). This approach seeks to unravel the intricate pathophysiology underlying multiple NCDs and understand their interaction with environmental and lifestyle factors. By leveraging the insights gained from the TREND cohort, we aspire to enhance preventive and therapeutic strategies, offering novel perspectives to advance both public health initiatives and clinical management for NCDs.

## Cohort Design

### Participants Recruitment

This cohort initiative aims to recruit 20,000 residents of Ma'anshan over a three-year period encompassing three phases. The city of Ma'anshan positioned at coordinates 31.67°N and 118.51°E in the south of Anhui province, China. This cohort will include all parts of Ma'anshan (i.e., 3 districts and 3 counties). Inclusion criteria for participant recruitment involve: i) age over 18 years; ii) residence Ma’anshan for a minimum of one year, with a minimum stay of nine months annually; iii) likely to stay in Ma’anshan for the foreseeable future and willing to participate; iv) able to provide written informed consent; v) capable of communicating with the research team.

The first-phase, involving 3360, took place during August–October 2023. The initial two days served as a pilot phase with a total of 240 participants aged 18–85 years. After the pilot phase, measurement tools and protocols underwent revision to improve the validity and reliability of the study, as well as to streamline data collection. This iterative process resulted in modification of the questionnaires and the procedures for collecting clinical measurements, biological samples and anthropometry.

The TREND cohort used a careful approach in participant selection, as outlined in the Supplementary Methods. To increase the feasibility of the study, individuals are recruited from both urban and rural areas. The allocation of the participants is proportional to the overall population covered by each health center, also referred to as research center.

A rigorous selection process is applied to all study personnel, including questionnaire investigators, laboratory technicians and executive managers, who underwent face-to-face interview and well comprehensive training.

Details of household members, including name, gender, age and relationship, are carefully registered, along with their contact number. When two household members meet the inclusion criteria and consent to participate, an informative pamphlet outlining the research plan, methods and standard conditions of biological sample collection will be given. In addition, sterilized kits for biological sample collection are provided. A personal invitation is extended, and a scheduled date for attendance at the respective health center is arranged.

In case where individuals are unreachable after three attempts, qualified replacements are sought. A reminder call is made one day prior to the scheduled physical examination appointment. Participants are advised to fast for at least 8 h before attending the health center. Upon arrival, receptionists confirm that the person meet the inclusion criteria and record any morning medication use. Each participant is assigned a unique digit code for identification purposes. Following enrolment, participants attend the clinical laboratory for physical examination and blood sample collection.

### Follow-up Visits

**Fig. 1:**
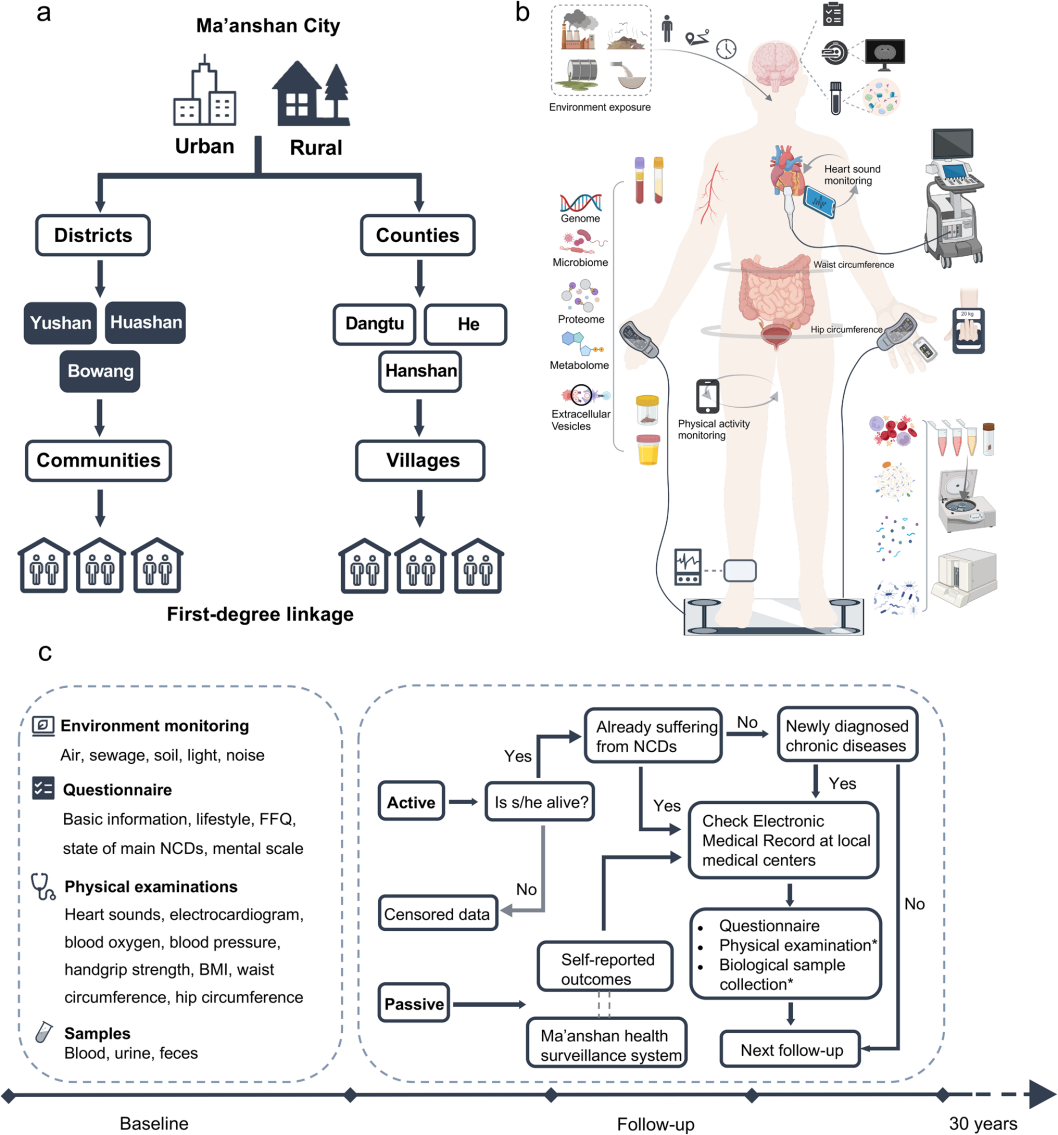
Study design and work scheme of the TREND cohort. **a** The TREND cohort is based in Ma'anshan, Anhui Province, China, including all six parts (i.e., three districts and three counties), where participants originated from urban and rural area with two first-degree-linkage family members of the same household will be recruited; **b** Deep phenotyping, molecular profiling and mHealth are undertaken per participant; **c** Data collected at baseline includes environment monitoring, questionnaire, physical examinations, and biological samples, with annual and interval follow-up for 30 years. *FFQ* food frequency questionnaire, *NCDs* Non-communicable Diseases

During the follow-up phase, study participants are regularly followed up for outcomes that primarily related to NCDs (e.g., cardiovascular diseases, metabolic dysfunction, cancer, respiratory diseases, degenerative diseases, and psychiatric diseases). This involves annual face-to-face or phone interviews, and additional interviews after the occurrence of any relevant event. Data, samples and measurements collected during the recruitment phase are systematically recollected at intervals of 5, 10, 15, 20, 25, and 30 years after the baseline. For participants experiencing an event of interest, key biological samples are promptly collected. The follow-up process incorporates both active and passive modes. Active follow-up is conducted by trained investigators, often doctors or nurses from each health center, who contact participants annually. Passive follow-up involves collecting self-reports when participants visit the health center to report events and utilizes the Ma'anshan health surveillance system, including data from disease and death registries (see Fig. 1).

If an event of interest occurs, the outcome review forms are completed by the certificated doctors. Live participants with confirmed outcomes are invited to the health center for further investigation. To mitigate recall bias, participants are asked to provide relevant medical documents. The reviewing investigators assess the recorded data, follow-up questionnaires, laboratory results, available biological samples and other medical documents (e.g., death certificate) to confirm the diagnosis.

Participation concludes if the person becomes unwilling to continue participation, faces difficulties reading or recalling information, migrates, becomes inaccessible, or dies. In case of unwillingness to continue, the follow-up team reinvites the participants for investigating the reasons and emphasizing the benefits of participation in the research. In addition, valuable advice for NCDs prevention will be provided.

## Measurement Indicators

### Questionnaire Survey

Participants undergo a comprehensive assessment through an interviewer-administered questionnaire conducted in Mandarin, with additional clarification provided in local dialects when needed, familiar to both the participant and interviewer. The questionnaire, comprising 898 items (refer to Table S1), is an adapted version of a nationally validated questionnaire for non-communicable diseases (NCDs). This questionnaire consists of both household- and individual items, covering general information, lifestyle, medical history, dietary intakes, and mental health evaluation. The household items focus on evaluating socio-economic status, family structure and monthly seasoning use for the entire family. The individual questionnaire includes demographic characteristics, personal and family medical history and lifestyle behaviors such as alcohol consumption, sedentary behavior, cigarette smoking and exposure to environmental tobacco smoke. All participant medications used are meticulously recorded.

Dietary intakes are assessed using a semi-quantitative food frequency questionnaire (FFQ) with 106 main items, followed by a series of sub-items, designed based on well-known cohorts (Bycroft et al. 2018; Jiao et al. 2023; Schram et al. 2014; Sun et al. 2022) and the national NCDs questionnaire, with considerations for local lifestyle and dietary patterns. Beyond the FFQ, eating habits, changes in dietary patterns, consumption of ultra-processed foods (UPF) and take-away food order are also assessed. The amount of dietary intakes is determined by investigators based on participants descriptions, using a graphical food amount illustration pamphlet for estimation.

The Physical Activities Questionnaire, incorporating 29 specific items, is revised according to the International Physical Activity Questionnaire Short Form (IPAQ-SF) (Lee et al. 2011). Variables such as weekly physical activity level (MET-h/week) and the intake of macronutrients, micronutrients, and daily servings by food groups are derived from the physical activity questionnaire and FFQ.

Assessment of sleep quality and mental health involves validated questionnaires, including Pittsburgh Sleep Quality Index (PSQI) (Mollayeva et al. 2016), Short Form 36 (SF-36) (Ware 2000), Zung Self-Rating Depression Scale (SDS) (Biggs et al. 1978), Sport Anxiety Scale (SAS) (Smith et al. 1990), and Mini-Mental State Examination (MMSE) (Mitchell 2013). This comprehensive approach ensures a thorough understanding of participants’ health, lifestyle, and psychological well-being.

### Physical Examination

Upon arrival at the health center participants undergo a thorough physical examination encompassing key anthropometric measurements. These measurements include height, waist circumference, hip circumference, weight, and body composition. To ensure accuracy, participants are instructed to remove shoes and objects from their pockets before standing on a digital stadiometer for the measurement of height and weight. A stretch-resistant tape will be used to measure waist circumference at the mid-point between the last rib and iliac crest, while hip circumference at the greater trochanter of the femur, both assessments will be performed with participants donning lightweight clothing. The evaluation of body composition, including adipose tissue, lean tissue, muscle tissue, water, protein, and mineral, will be conducted with participants attired in light clothing and without shoes, adhering with prescribed operating instructions. To ensure accuracy, multiple measures will be taken, and the subsequent analysis utilizes the average of these measures.

A comprehensive cardiovascular health examination will be conducted both at rest and during a handgrip test. Resting measurements include blood oxygen levels, electrocardiogram (ECG), and heart sound recordings. A newly-designed program, utilizing the built-in microphone of smartphones, facilitates the recording of heart sounds. Subsequently, signal processing algorithms filter the recorded heart sounds to eliminate background noises, allowing participants to listen to the clarified recordings. The handgrip test assesses participants' cardiac response to exercise, with heart sounds measured during the test. Blood pressure will be measured immediately before and after the handgrip test, providing valuable insights into cardiovascular health.

### Biological Samples

Participants are instructed to fast for at least 8 h before their second-morning visit to the health center. The collected blood samples consist of one 10-ml tube and one 5-ml tube, both anticoagulated with ethylenediaminetetraacetic acid (EDTA), along with two tubes for serum separation. Among these one 10-ml tube and one 2-ml tube are prepared with gel separation.

A total of 30 ml mid-stream morning urine is collected for routine urine substances analysis. Following timed and protocolled coagulation, the tubes designed for serum separation, as well as the blood and urine samples, are stored at 4 °C and transported from the laboratory under tightly controlled and continuously monitored conditions.

A portion of the samples is directly transferred to the local clinical laboratory for routine clinical chemistry assays on fresh samples. The remaining blood sample undergo centrifuged to separate substances such as plasma, serum, blood cells, and buffy coat. These blood components, along with the urine samples are aliquoted and stored at −80 °C, ensuring preservation for future research endeavors.

Fecal samples are collected at home, immediately stored at 4 °C, and promptly transferred to the laboratory. Three types of fecal aliquots are preserved, including raw feces, feces mixed with an RNA stabilizer, and feces mixed with 50% concentration of mixture of glycerin and cysteine. These fecal samples undergo aliquoted and stored at −80 °C until further processing.

In the baseline analysis, a comprehensive set of routine measurements are applied to evaluate various physiological aspects. The assessments cover blood-related parameters, including routine blood examination, glycemia-related indicators, lipid-related measures, hepatic and kidney function, myocardial zymogram, homocysteine levels, tumor markers, thyroid function, hepatitis markers, trace elements, immune and inflammatory markers, and other parameters that provide insights into physiological functionalities. For urine analysis, parameters such as urine specific gravity, urine pH, urine proteins, urine glucose, urinary ketone bodies, urine occult blood, urobilinogen, and urine bilirubin are examined. Fecal analysis includes cell type counts, fecal occult blood, and the presence of parasitic worms, as detailed in Table S2.

This thorough baseline analysis aims to establish a comprehensive understanding of participants' health status by assessing a wide range of physiological parameters across different bodily systems, contributing valuable data for the study.

### Assessment of Exposure to Environmental Pollution

Participants' residential details are collected from their National Identity Card, including information about any address changes and duration spent at each residence during both the baseline investigation and follow-up. The addresses are then converted into standardized longitudes and latitudes. Utilizing the Ma'anshan environmental surveillance system, spatial distributions of average pollutant levels (e.g., PM_2.5_, PM_10_, SO_2_, NO_2_, CO, O_3,_ sewage, noise, light, and soil) on different days are interpolated. Subsequently, the system calculates each participant's average exposure to these pollutants. The final exposure is determined by averaging exposures at all addresses, providing a comprehensive assessment of participants' average exposures to various pollutants during distinct exposure periods.

### Follow-up Plan

For the annual follow-up, in addition to updating the participant's address and contact details, comprehensive information on their medical history from the preceding year will be gathered. This includes any hospital admission, treatment received, and noteworthy chances that transpired. However, for the 5-year interval follow-up, our intention is to repeat the thorough assessment conducted during the recruitment phase.

### Quality Control and Data Processing

Interviewers and personnel conducting physical examination undergo rigorous training and competency assessment before commencing data collection. This ensures standardized interviewing and consistency in both physical examination and sample collection. To verify data accuracy, 10% of the completed questionnaires undergo a sampling process, and all data forms, including consent forms, undergo scrutiny for missing information. Two independent staff members perform double data entry, with any discrepancies resolved by the principal investigators through verification with source documents. After data entry completion, forms are optically scanned and archived. Research data are de-identified, key-coded and stored in research databases accessible only to database administrators, who conduct additional data cleaning by checking for consistency between related variables and data ranges before releasing the data for research analysis. Personal participants information and study visit records are stored separately and accessible only to the fieldwork management team for follow-up purposes.

## Key Findings

In the first phase study, 3352 individuals were recruited, with 3312 undergoing physical examinations. Basic characteristics of the participants are summarized in Table S3. Of the participants, 1998 (59.6%) were women, with a mean age was 59.82 years old for men and 55.31 years old for women. Educational attainment showed that 26.3% completed middle school, while 44.1% obtained a high school degree or higher.

The majority of participants (80.8%) were married, and the occupational categories were variously distributed. Among men, 38.1% were current smokers, and 6.6% were former smokers. For women, 1.0% were current smokers, and 0.3% were former smokers. Rates of alcohol use in the past year were 38.7% and 6.2% for men and women, respectively. The mean value for body mass index (BMI), waist circumference, and hip circumference were 23.9 kg/m^2^, 84.3 cm, and 94.5 cm, respectively. Based on the Working Group on Obesity in China criteria (Pan et al. 2021), the prevalence of overweight (BMI ranging from 24 kg/m^2^ to 28 kg/m^2^) and obesity (BMI ≥28 kg/m^2^) were 39.6% and 18.3%, respectively.

The self-report prevalence of hypertension was 24.9% for men and 22.3% for women. Similarly, the prevalence of diabetes mellitus and cancer was higher in men compared to women (diabetes mellitus prevalence: 12.8% vs. 10.0% and cancer prevalence: 1.5% vs 1.3%). Approximately 45.1% participants reported a family history of NCDs (i.e., at least one family member with at least one NCD), with hypertension (23.4%) being the most common disease with family aggregation, followed by diabetes mellitus (11.1%) and hyperlipidemia (6.4%).

Table S4 presents lifestyle, anthropometric measures, and biochemical characteristics of the participants. The mean systolic blood pressure and diastolic blood pressure were 127.5 mmHg, and 82.2 mmHg, respectively. The mean fasting blood glucose, HbAc1, insulin, triglycerides (TG), high-density lipoprotein cholesterol (HDL-C) and low-density lipoprotein cholesterol (LDL-C) were 5.7 mmol/L, 5.4%, 11.8 mIU/L, 1.8 mmol/L, 1.4 mmol/L and 3.1 mmol/L, respectively. In addition, the measured variables of blood routine results, hepatic function, kidney function, and urine parameters are presented in Table S2.

Using the Short Form 36 version 2 (SF-36v2) questionnaire (Ware 2000), we found that men had higher physical health component summary scores than women, while women demonstrated higher mental health component summary scores than men. Cognitive abilities were assessed across five cognitive domains (MMSE (Mitchell 2013)): memory, orientation, arithmetic function, executive function, and object naming. Gender differences in the cognitive score were pronounced among the elderly aged >65 years; 8.2% of elderly men were found in the lowest 10th percentile of composite cognition scores compared with 19.3% of elderly women.

The quality control (QC) assessment for collected data was available for 335 participants (10%). The comparison between the baseline and QC investigation revealed strong agreement for various variables, with kappa coefficients being 0.81 for smoking status and 0.74 for alcohol consumption. Height, weight, and waist circumference demonstrated robust within-person Spearman correlation coefficients of 0.97, 0.98, and 0.94, respectively, indicating a high level of concordance between the baseline survey and the QC investigation.

## Strengths and Limitations

This cohort boasts several notable strengths, primarily stemming from its comprehensive approach to phenotyping coupled with the inclusion of diverse lifestyle determinants that have the potential to impact the initiation and progression of health challenges in individuals. The integration of multi-omics, mHealth, lifestyle and environmental data, combined with extensive clinical phenotyping and long-term follow-up of TREND participants, enhances the capacity to pinpoint early-stage NCD development. Moreover, this cohort plays a crucial role in unraveling novel mechanistic biomarkers and pathways, offering a better and deeper understanding of the transition from health to disease and the various disease states. Ultimately, it aids in tailoring disease-specific therapies based on individual bio-profiles.

The first-degree linkage design employed in this study allows for the exploration of family-specific genetic background that may influence NCDs. Simultaneously, this study design enables the evaluation of gene-environment interactions by examining whether an environmental risk factor exerts distinct effects on individuals with a genetically higher risk, identified through familial aggregation, compared to those at a lower risk.

The first phase of the study has provided crucial support for researchers to assess the feasibility of various data collection aspects and validate new instruments withing our specific context. The comprehensive nature of this study, encompassing detailed assessments and the establishment of a biobank, will be instrumental in facilitating a broad spectrum of research endeavors in the future.

One limitation, prevalent in numerous prospective cohort studies, stems from the reliance on self-reported data, potentially introducing a degree of unreliability. The efficacy of longitudinal cohort studies heavily hinges on participant commitment to recruitment and follow-up, making the TREND susceptible to selection bias due to its single-center nature, nonparticipation, and loss to follow-up, thereby restricting generalizability to other populations. Additionally, individuals willing to engage in long-term research might exhibit heightened health consciousness and adopt specific lifestyles, introducing a source of bias. Despite measuring numerous parameters, the study acknowledges the potential impact of unmeasured or residual factors, which cannot be entirely eliminated.

## Supplementary Information

Below is the link to the electronic supplementary material.

Supplementary file1 (DOCX 41 KB)

## Data Availability

Access to individual-level data from the TREND cohort is available for research and scientific purposes, provided with the informed consent agreed upon by the TREND participants. This consent explicitly states that the collected samples and data will not be used for commercial purposes. Researchers seeking access to individual-level data must secure approval from the management board of the TREND cohort and adhere to the policies established by the Human Genetic Resource Administration, Ministry of Science, and Technology of the People’s Republic of China. To maximize the use of data and biospecimens, the TREND study welcomes and encourages global collaborations. Researchers interested in collaboration can reach out via E-mail to L.M.Chen [lianminchen@njmu.edu.cn] or E.Y.W.Yu. [evan.yu@maastrichtuniversity.nl].
