# Supplementary material for: Cohort Profile: TRacing Etiology of Non-communicable Diseases (TREND): Rationale, Progress and Perspective
Source: Phenomics. 2024 Oct 16;4(6):584–91. doi: 10.1007/s43657-024-00196-4 (PMC11889304; doi:10.1007/s43657-024-00196-4)
Supplement: Supplementary file 1 — Supplementary file1 (DOCX 41 KB) [file 43657_2024_196_MOESM1_ESM.docx]

Supplementary File to **“Cohort Profile: TRacing Etiology of Non-communicable Disease (TREND): Rationale, Progress and Perspective”**

Hui-Ying Ren ^#^, Ying Lv ^#^, Bei-Ning Ma ^#^, Chang Gao, Hong-Mei Yuan, Hai-Hong Meng, Zheng-Qian Cao, Ya-Ting Chen, Yan-Xi Zhang, Yu-Ting Zhang, Wei Liu, Yu-Ping Fan, Meng-Han Li, Yu-Xuan Wu, Zhuo-Yue Feng, Xin-Xin Zhang, Zhen-Jian Luo, Qiu-Yi Tang, Anke Wesselius, Jian Chen, Hong-Xing Luo, TREND Cohort Study, **Qi-Rong Qin ^*^**, **Lianmin Chen ^*^**, **Evan Yi-Wen Yu ^*^**

*Correspondence to:

**Evan Yi-Wen Yu, PhD**

E-mail address: [evan.yu@maastrichtuniversity.nl](mailto:evan.yu@maastrichtuniversity.nl)

**&**

**Lianmin Chen, PhD**

E-mail address: lianminchen@njmu.edu.cn

**&**

**Qi-Rong Qin, PhD**

E-mail address: qqr2022@163.com

**Supplementary Methods and Materials**

Using the Ma’anshan Demographic database, a multi-stage stratified sampling approach is employed to ensure the selection of a representative cohort population. The sample size for each district or county was set at 3,360, which was determined according to the average prevalence with at least one chronic disease of adults in China reported up to 2022, taking into account the linkage design per household. The sampling process is delineated as follows:

**Stage I;**

Available health centers (research centers) are summarized at first, then the number of communities or villages is determined according to the population proportion of each health center with consideration of the locations accounting for northern, southern, eastern, western and central directions. At least 60 households, i.e., 120 residents, are randomly recruited according to the inclusion criteria in the selected communities or villages.

**Stage II;**

The number of households to be recruited are implemented by the administrators of each research center, while the shortfall of households who meet all the inclusion criteria will be compensated by neighboring communities or villages. When the number of households of the residents is greater than the number of households required for the study, the participants are randomly selected. The principle of two participants per household are incorporated into the process, with at least 60 households being recruited in each of selected community or village at this stage.

**Stage III;**

Among the selected households, two participants with first-degree linkage, taking differences of age and gender into account, are recruited. The ideally principle is to recruit parents-offspring, followed by grandparents-offspring or siblings.

**Special principle of participants recruitment;** i) Migration of households: in the scenario of a recruited household relocating, recruitment should be investigated based on the new household that has moved in; ii) Rental housing: if the residence of the recruited household has been rented out, and the actual owner does not reside in this house, the principle is to investigate the individuals who currently live in this house; iii) in cases where more than one family reside in a household, a family should be identified for investigation based on the principle of proximity to the entry door followed by distance.

**Replacement of recruited households;** i) Replacement of participants: if the recruited individuals are not at home or is extremely uncooperative for at least 3 times, this household will then be replaced; ii) Replacement method: according to the location of the original households, the order of choosing the appropriate residents to replace the household are from left to right, up to down, inside to outside.

**Table S1** Summary of variables collected or derived from baseline evaluation of the first phase study

| **Characteristics** | **Labelling** | **Description** |
| --- | --- | --- |
| **General** |  |  |
|  | Demographic characteristics | Familial relationship, gender, age, ethnicity, educational levels, marital status, occupation, health insurance |
|  | Socio-economic factors | Household income, household expenditure, access to facilities of health |
|  | Fuel use and living standards | Types of cooking fuel, heating system, living status, kitchen status, family structure |
|  | Lifestyle exposures | Smoking (including active smoking, passive smoking, smoking status, smoking intensity, smoking duration, e-cigarette information), alcohol consumption (including alcohol subtypes, frequency and amount of consumption), tea consumption (including tea subtypes, frequency and amount of consumption) |
|  | Physical activity | International Physical Activity Questionnaire (IPAQ), including exercise status, job activities, home activities, professional sports activities, metabolic equivalent, sleep status, rest status |
|  | Sleep status | Pittsburgh Sleep Quality Index (PSQI), including night sleep status, daytime nap, night shift work, sleep problems, sleeping pills use |
|  | Specific characteristics for women | Menstruation, pregnant history, history of giving birth, screening history for breast cancer and cervical cancer, HPV vaccination |
| **Medical** |  |  |
|  | Past medical history | Medical condition diagnosis, start age of medical condition, medical condition treatment |
|  | Sign and symptoms | Abnormal signs and symptoms of various organs of the body, fracture status, history of surgery, hospitalization status, allergy history and symptoms |
|  | Medication use | Past and present medication use, including oral, injection medication use, vaccination for preventable purpose |
|  | Family Medical History | Disease in first-degree relatives, second-degree and third-degree relatives |
|  | Reproductive history (women) | Menstruation status, pregnancy status, stillbirth, abortion, breastfeeding, menopause, cervical or breast screening, HPV vaccination |
|  | Oral health | Brushing, teeth status, oral lesion, flossing, dentures, mouthwash |
|  | Mental health | Sport Anxiety Scale (SAS), Zung Self-Rating Depression Scale (SDS), SDS, MMSE |
|  | Knowledge about NCDs | Participants’ prior knowledge about NCDs |
|  | Health service | Physical examination, advice and help obtained from local health center |
| **Nutrition** |  |  |
|  | Household seasoning use | Amount use of salt, added sugar, oil, monosodium glutamate, soy sauce and other sauce per month |
|  | Food frequency questionnaire (FFQ) | Amount and frequency of consumption of different types of food, local foods, nutrients and water consumption |
|  | Dietary habits | The number of meals usually eaten in a day, type of cooking ware, eating habit change, ultra-processed food, months of intakes, herbal use, eating flavor |
|  | Dietary supplement intake | Daily intake of various dietary supplements |
| **Anthropometry** |  |  |
|  | Anthropometric indicators | Height, weight, waist circumference, hip circumference, BMI, body composition, ECG, blood oxygen, and heart sound |
| **Biological samples** |  |  |
|  | Biobank samples and lab tests | Urine, blood, fecal sample; physical, chemical and microscopic examination of urine sample, biochemistry and hematology analysis of blood sample, microscopic examination of fecal sample |

**Abbreviations:** IPAQ, International Physical Activity Questionnaire; SAS, Sport Anxiety Scale; SDS, Zung Self-Rating Depression Scale; MMSE, Mini-Mental State Examination; NCD, non-communicable disease; BMI, Body Mass Index; ECG, electrocardiogram.

**Table S2** Summary of variables of baseline laboratory measurements of the first phase study

| **Characteristics** | **Labelling** |
| --- | --- |
| **Routine blood test** |  |
|  | Red blood cell count (10^12^/L) |
|  | White blood cell count (10^9^/L) |
|  | Platelet count (10^9^/L) |
|  | Hemoglobin (g/L) |
|  | Neutrophil count (10^9^/L) |
|  | Lymphocyte count (10^9^/L) |
|  | Monocyte count (10^9^/L) |
|  | Eosinophil count (10^9^/L) |
|  | Basophil count (10^9^/L) |
|  | Neutrophil percent (%) |
|  | Lymphocyte percent (%) |
|  | Monocyte percent (%) |
|  | Eosinophil percent (%) |
|  | Basophil percent (%) |
|  | Hematocrit (%) |
|  | Mean Corpuscular Volume (MCV; fL) |
|  | Mean Corpuscular Hemoglobin (MCH; pg) |
|  | Mean Corpuscular Hemoglobin Concentration (MCHC; g/L) |
|  | Platelet Crit (PCT; %) |
|  | Mean Platelet Volume (MPV; fL) |
|  | Platelet Distribution Width (PDW; %) |
|  | Red Cell Distribution Width - Coefficient of Variation (RDW-CV; %) |
|  | Red Cell Distribution Width - Standard Deviation (RDW-SD; fL) |
| **Glycemia-related parameters** |  |
|  | Blood glucose (mmol/L) |
|  | HbA1c (%) |
|  | Insulin (mIU/mL) |
|  | Fructosamines (mmol/L) |
| **Lipid-related parameters** |  |
|  | Triglycerides (mmol/L) |
|  | Total cholesterol (mmol/L) |
|  | High-density lipoprotein cholesterol (HDL-C; mmol/L) |
|  | Low-density lipoprotein cholesterol (LDL-C; mmol/L) |
|  | ApoA1 (mmol/L) |
|  | ApoB (mmol/L) |
|  | Lipoprotein (a) (mg/L) |
| **Hepatic function** |  |
|  | Alanine Aminotransferase (ALT; U/L) |
|  | Aspartate Aminotransferase (AST; U/L) |
|  | Alkaline Phosphatase (ALP) |
|  | Total Protein (TP; g/L) |
|  | Albumin (g/L) |
|  | Globulin (g/L) |
|  | Total Bilirubin (μmol/L) |
|  | Direct Bilirubin (μmol/L) |
|  | Indirect Bilirubin (μmol/L) |
|  | Total Bile Acids (μmol/L) |
| **Kidney function** | Serum Creatinine (μmol/L) |
|  | Urea (mmol/L) |
|  | Uric Acid (mg/L) |
| **Myocardial zymogram** |  |
|  | Gamma-Glutamyl Transferase (GGT; U/L) |
|  | Cholinesterase (U/L) |
|  | Alpha-L-fucosidase (mU/ml) |
|  | Lactate Dehydrogenase (U/L) |
|  | Lactate Dehydrogenase Isoenzyme (U/L) |
|  | Creatine Kinase (U/L) |
|  | Creatine Kinase-Muscle and Brain (U/L) |
| **Amino acid** |  |
|  | Homocysteine (μmol/L) |
| **Tumor markers** |  |
|  | Alpha-Fetoprotein (ng/mL) |
|  | Carcinoembryonic Antigen (ng/mL) |
| **Thyroid function** |  |
|  | Total Triiodothyronine (pmol/L) |
|  | Total Thyroxine (pmol/L) |
|  | Free Triiodothyronine (pmol/L) |
|  | Free Thyroxine (pmol/L) |
|  | Thyroid-Stimulating Hormone (pmol/L) |
| **Hepatitis infection** |  |
|  | HbsAg (IU/mL) |
|  | HbsAb (IU/mL) |
|  | HbeAg (IU/mL) |
|  | HbeAb (IU/mL) |
|  | HbcAb (IU/mL) |
| **Trace element** |  |
|  | Ca (mmol/L) |
|  | Mg (mg/dL) |
|  | P (mg/dL) |
| **Inflammatory parameters** |  |
|  | C-Reactive Protein (mg/L) |
|  | High-Sensitivity C-Reactive Protein (mg/L) |
|  | Procalcitonin (ng/mL) |
| **Routine urine test** |  |
|  | Nitrite (μmol/L) |
|  | Urobilinogen (μmol/L) |
|  | Bilirubin（mg/dL） |
|  | Specific gravity |
|  | Protein（g/L） |
|  | Ketone（mmol/L） |
|  | Occult blood |
|  | White blood cell count (10^9^/L) |
|  | Urine pH |
| **Routine fecal test** |  |
|  | White blood cell count (10^9^/L) |
|  | Occult blood |

**Abbreviations:** MCV, Mean Corpuscular Volume; MCH, Mean Corpuscular Hemoglobin; MCHC, Mean Corpuscular Hemoglobin Concentration; PCT, Platelet Crit; MPV, Mean Platelet Volume; PDW, Platelet Distribution Width; RDW-CV, Red Cell Distribution Width-Coefficient of Variation; RDW-SD, Red Cell Distribution Width - Standard Deviation; HDL-C, High-density lipoprotein cholesterol; LDL-C, Low-density lipoprotein cholesterol; HbA1c, hemoglobin A1C; ApoA1, Apolipoprotein A1; ApoB, Apolipoprotein B; ALT, Alanine Aminotransferase; AST, Aspartate Aminotransferase; ALP Alkaline Phosphatase; TP, Total Protein; GGT, Gamma-Glutamyl Transferase.

**Table S3** Demographic profile of participants at baseline of the first phase study

| **Characteristics** | **Number** | **Percentage (%)** |
| --- | --- | --- |
| **Ethnicity** |  |  |
| Han Chinese | 3,339 | 99.6 |
| Other ethnicities | 13 | 0.4 |
| **Age at recruitment (years)** |  |  |
| 18–45 | 1,103 | 32.9 |
| 45–55 | 744 | 22.2 |
| 55–65 | 694 | 20.7 |
| ≥ 65 | 811 | 24.2 |
| **Sex** |  |  |
| Women | 1,998 | 59.6 |
| Men | 1,354 | 40.4 |
| **Marital status** |  |  |
| Never married | 295 | 8.8 |
| Currently married | 2,708 | 80.8 |
| Cohabitation | 17 | 0.5 |
| Divorce | 218 | 6.5 |
| Married but live apart | 97 | 2.9 |
| Widowed | 4 | 0.1 |
| Others | 13 | 0.4 |
| **Educational level** |  |  |
| No formal qualification | 322 | 9.6 |
| Primary | 670 | 20.0 |
| Secondary | 882 | 26.3 |
| Post-secondary | 597 | 17.8 |
| Vocational training | 479 | 14.3 |
| University and above | 402 | 12.0 |
| **Occupation** |  |  |
| Employed population of farming, forestry, husbandry, fishing and water conservancy industry | 211 | 6.3 |
| Employed population of production and transportation | 352 | 10.5 |
| Employed population of commerce, service trade Personnel | 426 | 12.7 |
| Employed population of governments agencies, Party agencies and social organizations | 184 | 5.5 |
| Employed population of professional/ technical | 392 | 11.7 |
| Unemployment | 235 | 7.0 |
| Non-working population-students in school | 47 | 1.4 |
| Non-working population-household affairs | 476 | 14.2 |
| Non-working population-retired | 657 | 19.6 |
| Others | 372 | 11.1 |
| **Yearly household income (CNY)** |  |  |
| ≤ 30 000 | 352 | 10.5 |
| 30 000–100 000 | 1,314 | 39.2 |
| 100 000–200 000 | 875 | 26.1 |
| 200 000–300 000 | 80 | 2.4 |
| > 300 000 | 17 | 0.5 |
| Prefer not to answer | 714 | 21.3 |

Categorical statistics are presented as frequency and percentage (%).

The occupation categories are listed according to China occupational classification (version 2022).

**Abbreviations:** CNY, Chinese Yuan.

**Table S4** Lifestyle, anthropometric measures, self-reported diseases, and biomarkers profiles of study participants at baseline of the first phase study

| **Characteristics** | **Men** | **Women** |
| --- | --- | --- |
| **Cigarette smoking status (%)** |  |  |
| Never smoker | 749 (55.3) | 1,972 (98.7) |
| Former smoker | 89 (6.6) | 6 (0.3) |
| Current smoker | 516 (38.1) | 20 (1.0) |
| **Alcohol Consumption (in the past year)** |  |  |
| No | 830 (61.3) | 1,874 (93.8) |
| Yes, ≤ 30 days | 402 (29.7) | 74 (3.7) |
| Yes, > 30 days | 122 (9.0) | 50 (2.5) |
| **Physical activity (mins/day;** **quartile 25–quartile 75)** |  |  |
| Heavy at work | 120 (60–210) | 120 (60–180) |
| Moderate at work | 60 (60–120) | 60 (60–120) |
| Moderate at entertainment and sports | 60 (30–70) | 60 (30–80) |
| Sedentary | 210 (120–300) | 180 (120–300) |
| **BMI category (kg/m^2^; %)** |  |  |
| ＜ 18.5 | 30 (2.2) | 60 (3.0) |
| 18.5–24.0 | 437 (32.3) | 881 (44.1) |
| 24.0–28.0 | 588 (43.4) | 741 (37.1) |
| ≥ 28.0 | 299 (22.1) | 316 (15.8) |
| **Blood pressure (mmHg), mean ± SD** |  |  |
| Systolic | 127.8 ± 18.7 | 128.0 ± 19.7 |
| Diastolic | 82.3 ± 10.9 | 82.1 ± 10.9 |
| **Self-reported diseases (%)** |  |  |
| Hypertension | 337 (24.9) | 446 (22.3) |
| Diabetes mellitus | 173 (12.8) | 200 (10.0) |
| Hyperlipidemia | 89 (6.6) | 126 (6.3) |
| Cancer | 20 (1.5) | 26 (1.3) |
| **Biomarkers, mean ± SD** |  |  |
| Fasting blood glucose (mmol/L) | 5.8 ± 2.0 | 5.7 ± 1.5 |
| HbA1c (%) | 5.5 ± 1.1 | 5.4 ± 1.0 |
| Insulin (mIU/mL) | 12.4 ± 11.6 | 11.5 ± 12.0 |
| Fructosamines (μmol/L) | 237.0 ± 40.0 | 237.4 ± 36.2 |
| Triglycerides (mmol/L) | 2.1 ± 2.1 | 1.6 ± 1.1 |
| High-density lipoprotein cholesterol (HDL-C) (mmol/L) | 1.3 ± 0.3 | 1.5 ± 0.3 |
| Low-density lipoprotein cholesterol (LDL-C) (mmol/L) | 3.1 ± 0.8 | 3.1 ± 0.9 |
| ApoA1 (mmol/L) | 1.5 ± 0.2 | 1.6 ± 0.2 |
| ApoB (mmol/L) | 1.1 ± 0.2 | 1.0 ± 0.3 |
| Lipoprotein (a) (mg/L) | 32.9 ± 50.9 | 44.2 ± 61.6 |

Data are shown as mean ± stand deviation for normal distribution continuous variables or median (quartile 25–quartile 75) for non-normal distribution continuous variables and frequncies (%) for categorical variables.

**Abbreviations:** mins, minutes; SD, standard deviation; HbA1c, hemoglobin A1C; ApoA1, Apolipoprotein A1; ApoB, Apolipoprotein B.
